# Psychometric Properties of the Chinese Version of the Premonitory Urge for Tics Scale: A Preliminary Report

**DOI:** 10.3389/fpsyg.2021.573803

**Published:** 2021-09-27

**Authors:** Ying Li, Douglas W. Woods, Yi Gu, Liping Yu, Junjuan Yan, Fang Wen, Fang Wang, Jingran Liu, Yonghua Cui

**Affiliations:** ^1^Department of Psychiatry, National Center for Children's Health, Beijing Children's Hospital, Capital Medical University, Beijing, China; ^2^Department of Psychology, Marquette University, Milwaukee, WI, United States

**Keywords:** premonitory urge, tic disorders, PUTS, psychometric properties, network analysis

## Abstract

Premonitory urges (PUs) are sensory phenomena that immediately precede tics. The Premonitory Urge for Tics Scale (PUTS) is widely used to assess the severity of PUs, but the psychometric properties of PUTS and clinical features of PU in Chinese patients with tic disorders are still unclear. In this study, we examined the psychometric properties of the Chinese version of the PUTS in a large sample (including 367 Chinese pediatric patients with tic disorders). We found no difference in PU in different age groups. The exploratory factor analysis (EFA) of PUTS showed the emergence of four primary factors. The results of reliability and validity analyses indicated that the Chinese version showed good psychometric properties. It seemed that PU was associated with the severity of obsession symptoms in patients with tic disorders. Network analysis showed that Item 7 is a critical node for the PU, in addition to Items 1 and 4. Overall, the Chinese version of PUTS can be used in Chinese child and adolescent patients with tic disorders, particularly for patients with Tourette syndrome.

## Introduction

Premonitory urges (PUs) are sensory phenomena that immediately precede tics (Conceicao et al., 2017). PUs include both sensory feelings (i.e., itch or pressure in certain bodily areas) and mental phenomena (i.e., an uncountable feeling of “not just right” or “incomplete”) (Cox et al., 2018). It is reported that over 90% of patients with Tourette syndrome (TS) experience PUs (Leckman et al., 1993). Previous meta-analysis research has shown a mild-to-moderate correlation between PU strength and tic severity (Wang et al., 2019). Although PU is often viewed as the earliest part of the tic movement process (Kyriazi et al., 2019), it has also been suggested that it is a separate and distinct symptom from the tic (Li et al., 2019). It has been reported that PU plays an important role in the tic generation (Rae et al., 2019). This has led some experts to recommend that the PU could be considered as a target symptom in reducing the tic symptoms (Nissen et al., 2019a,b).

How to assess the PUs? It was reported that the Premonitory Urge for Tics Scale (PUTS) was widely used for the assessment of PUs, which is a self-report checklist (Woods et al., 2005; Raines et al., 2018). Several studies have confirmed that PUTS shows good internal reliability (Raines et al., 2018; Openneer et al., 2020). The PUTS included nine items that assess both the sensory feelings and mental phenomena, and these “feelings” were mainly reported according to the self-awareness of patients (Ganos et al., 2015). However, it should be noted that some studies suggested that the culture might show potential influence on the psychometric properties of scales, especially for these self-report scales based on self-awareness (Dambi et al., 2018). It suggested that the psychometric properties of PUTS should be validated in a different culture. However, there is no Chinese version of the PUTS, and the psychometric properties of the Chinese version of PUTS have not been established.

In our previous report about the prevalence of tic disorder in China, it was 2.5% (Li et al., 2021). As screening tools for tic disorders play an important role in the early diagnoses of tic disorders, but only the Yale Global Tic Severity Scale (YGTSS) has been validated in the assessment of tic disorders in China (Wen et al., 2021). Moreover, behavioral therapy has been conducted in several hospitals in China (Sellbom and Tellegen, 2019), and awareness of PU is believed to play an important role in the efficacy of behavioral therapies, such as habit reversal training. However, there is no tool for the assessment of PUs currently in China.

Furthermore, Woods et al. (2005) suggested that the psychometric properties of the PUTS were not acceptable for youths 10 years of age and younger, which indicated that there might be an age effect of PUTS. However, a recent study found that the Cronbach's alfa (used for the assessment of internal consistency) was at the same level in two young groups (aged 3–7 and aged 8–10), but low Cronbach's alfa in the old group (aged 11–16, 0.76) (Openneer et al., 2020). In addition, Banaschewski et al. (2003) reported that there was no significant difference between the younger TS group aged 8–10 and the older group aged 11–14. Moreover, a recent study reported that PU showed a significant difference in different age groups (Openneer et al., 2020). It indicated that there might be an age effect on the reliability of PUTS and the severity of PU. However, we need further evidence to clarify these issues.

Besides, traditional analysis of psychometric properties for scales mainly focused on the differences of included items, such as the factor analysis (Sellbom and Tellegen, 2019). However, for the correlation of included items, the only correlation coefficient was calculated. Network analysis is a set of integrated techniques to depict relations among actors and to analyze the structures of scales that emerge from the recurrence of these relations (Contreras et al., 2019). It indicated that the network analysis can present the correlation of included items.

Therefore, this study aimed to measure the psychometric properties of the Chinese version of the PUTS in a Chinese setting. Specifically, we will investigate the reliability and validity of the Chinese version of the PUTS and perform a network analysis of PUTS to assess the structure of PU.

## Materials and Methods

### Participants

A total of 380 children and adolescents with tic disorders (based on the Diagnostic and Statistical Manual of Mental Disorders, Fifth Edition, DSM-5) participated in this research, 13 of them did not finish the whole survey and were subsequently excluded. Finally, a total of 367 patients between the ages of 6 and 16 years were identified. All of the participants were recruited from the Department of Psychiatry in Beijing Children's Hospital in China from October 1, 2019, to January 1, 2020. For the whole sample, we use the term “Total sample” to define it (*n* = 367). To assess the clinical characteristics of patients with TS, the term “TS group” (*n* = 252) was used to define patients with TS. In the consideration for the age effect of PUs, we will assess the validity and reliability of the PUTS in different age groups, the “Older group” (aged ≥ 10 years, *n* = 137) and the “Younger group” (aged <10 years, *n* = 230) were defined.

This study was approved by the ethics committees of Beijing Children's Hospital of Capital Medical University, and written informed consent was obtained from the guardians of the participants.

### Scales for Assessments

#### Premonitory Urge for Tics Scale

The PUTS is a nine-item self-report questionnaire measuring premonitory sensations in individuals with tics (Woods et al., 2005). Each item is scored from 1 (not at all true) to 4 (very much true). The total score is computed by summing the nine items. Total scores range from 9 to 36, where higher scores represent greater severity of PUs. The PUTS has demonstrated good internal consistency, test–retest reliability, and construct validity among adolescents between 11 and 16 years of age (Openneer et al., 2020).

Before carrying out the translation, we obtained an agreement from the authors of the original instrument (Woods et al., 2005). We then performed initial translation and forward–backward translation of the PUTS to Chinese (both Mandarin and Cantonese) according to the guidelines proposed by Beaton et al. (2000).

#### The Yale Global Tic Severity Scale

The YGTSS is a semi-structured interview developed for the assessment of the nature and severity of motor and vocal tics (Leckman et al., 1989; Wen et al., 2021). The assessment dimensions of the YGTSS include the number, frequency, intensity, complexity, and interference of vocal and motor tic symptoms, with a maximum score of 50 for tic severity (25 for motor and 25 for vocal tics) and a score of 50 for the impairment caused by the tics, yielding a total maximum score of 100. The YGTSS is a widely used scale with excellent psychometric properties (McGuire et al., 2018) and demonstrated excellent internal consistency (α = 0.91) in the present sample.

#### Children Yale-Brown Obsessive Compulsive Scale

The CY-BOCS is a 10-item clinician-rated measure of obsessive-compulsive disorder (OCD) symptom severity (Scahill et al., 1997). Raters assess obsessions and compulsions separately in five domains: time spent, interference, distress, resistance, and control. Each domain is rated on a scale from 0 to 4, and the total scores range from 0 to 40, with higher scores indicating greater symptom severity. The CY-BOCS has demonstrated good inter-rater reliability, internal consistency, and convergent and discriminant validity (Storch et al., 2006). However, there is no Chinese version of CY-BOCS. Three steps were performed before the assessment of CY-BOCS. First, we translated the CY-BOCS into Chinese. Second, the back-translation was performed to confirm that the meaning of each item of Chinses version CY-BOCS was the same as the original version of CY-BOCS. Third, in the present sample, the tool demonstrated excellent internal consistency (α = 0.89).

In addition, two psychiatrists were invited to perform the assessment of this study, and the intraclass correlation coefficient was >0.85. All the participants were outpatients. After the participants were diagnosed, the assessments were performed in the psychology assessment room in the Department of Psychiatry in Beijing Children's Hospital by one of the two psychiatrists.

### Statistical Analysis

Statistical analyses were performed using the Statistical Package for the Social Sciences for Windows (SPSS Inc., Chicago, IL, USA, v25.0). First, we compared PUTS, YGTSS, and CY-BOCS between the Younger group and the Older group using a *t*-test. Second, we examined the item-total correlation using bivariate correlations (Pearson's correlation). Third, we conducted an exploratory factor analysis (EFA) using principal component analysis on the PUTS items. Forth, we tested for internal consistency for factors and total scale using reliability analysis (Cronbach's alpha, Spearman-Brown, and test–retest reliability). Fifth, we determined external validation by correlating PUTS scores with the total score of YGTSS and CY-BOCS. Finally, network estimation was calculated by the “qgraph” package (Epskamp et al., 2012) in R software (Version 3.2.2; https://www.r-project.org/). We used the “Pcor” and “Cor” network models to regularize the correlation network (“Cor” network) to obtain the optimal network structure (Epskamp et al., 2018), and we calculated the centrality of each node (Strength, Closeness, and Betweenness) to represent the importance of each node in the network.

A “Cor” network is a model for marginal independence relationships; two unconnected nodes imply marginal independence between two nodes. The partial correlation network (“Pcor” network) is a model for conditional independence relationships. The “Pcor” network implies the opposite kind of independence relationships between unconnected nodes; unconnected nodes are conditionally independent but marginally still allowed to correlate (Epskamp et al., 2012). There were three measures (Strength, Closeness, and Betweenness) used to assess the weighted networks (Epskamp et al., 2018). Strength is a measure that takes into consideration the total level of involvement of a node in the network; Closeness is defined as the inverse of farness, which in turn, is the sum of distances to all other nodes; the extent to which a node is part of transactions among other nodes can be assessed by Betweenness (Epskamp et al., 2018).

## Results

### Clinical Characteristics Across the Groups

The mean age of included patients was 9.53 ± 2.06 years, and the mean duration of illness was 2.66 ± 1.61 years. About 8.7% of participants reported a positive family history of tics. The mean and SD of PUTS, YGTSS CY-BOCS for the different groups are presented in Table 1. Furthermore, to investigate the age effects of PU, we performed a *t*-test to compare the differences between the Younger group and the Older group. We found no difference in PUTS and YGTSS (*p* < 0.05), but there was a significant difference found in CY-BOCS (*p* = 0.013).

**Table 1:** The clinical characteristics for different groups.

**Groups**	***N***	**Male/female**	**Mean age (SD)**	**Total PUTS Mean (SD)**
Total sample	367	293/74	9.53(2.06)	12.95 (3.27)
TS group	252	204/48	9.6 (2.17)	13.19 (3.23)
Younger group	230	183/47	8.5 (1.30)	12.48 (3.01)
Older group	137	110/27	12.4 (1.39)	13.54 (3.51)
*P*-value (younger vs. older)	N/A	0.867	0.000	0.457

*PUTS, Premonitory Urge for Tics Scale; SD, Standard Deviation; N/A, Not Available; Younger Group: age <10; Older Group: age ≥ 10*.

### Item Correlations With the Total Score and the Results of EFA

We also calculated the correlation between each item and the nine-item total score. All items significantly correlated with the nine-item total score (*p* < 0.05; Table 2).

**Table 2:** Correlation between the Items Scores and Total PUTS Scores and Results of EFA.

**Items**	**Mean**	**SD**	**Skewness**	**Kurtosis**	**Correlation with PUTS total**	**Component 1**	**Component 2**	**Component 3**	**Component 4**
PUTS 1	1.26	0.551	2.256	5.191	0.265**	0.118	−0.056	0.032	0.956
PUTS 2	1.07	0.283	4.108	17.866	0.281**	0.052	0.879	0.039	−0.059
PUTS 3	1.10	0.370	4.792	27.821	0.342**	0.079	0.851	0.183	0.014
PUTS 4	1.72	0.838	0.880	−0.147	0.631**	0.811	−0.099	0.041	−0.335
PUTS 5	1.05	0.288	6.195	38.398	0.242**	0.043	0.265	0.773	−0.148
PUTS 6	1.07	0.323	4.792	23.521	0.367**	0.123	−0.008	0.807	0.152
PUTS 7	1.98	0.837	0.666	0.026	0.895**	0.926	0.113	0.069	0.199
PUTS 8	1.85	0.721	0.730	0.787	0.827**	0.911	0.128	−0.017	−0.335
PUTS 9	1.95	0.977	0.865	−0.203	0.785**	0.717	0.077	0.408	0.135

PUTS, Premonitory Urge for Tics Scale; SD, Standard Deviation; EFA, exploratory factor analysis; **p < 0.001. PUTS 1: Right before I do a tic I feel like my insides are itchy; PUTS 2: Right before I do a tic I feel pressure inside my brain or body; PUTS 3: Right before I do a tic I feel “wound up” or tense inside; PUTS 4: Right before I do a tic I feel like something is not “just right”; PUTS 5: Right before I do a tic I feel like something isn't complete; PUTS 6: Right before I do a tic I feel like there is energy in my body that needs to get out; PUTS 7: I have these feelings almost all the time before I do a tic; PUTS 8: These feelings happen for every tic I have; PUTS 9:After I do the tic, the itchiness, energy, pressure, tense feelings or feelings that something isn't “just right” or complete go away, at least for a while. Underline means the included items in this Component.

The results of the EFA showed that Kaiser-Meyer-Olkin (KMO) was 0.679, *p* < 0.001, indicating that the data were suitable for factor analysis. Four factors were extracted, including component 1 (Items 3 and 7, 8, and−9), component 2 (Items 2 and 3), component 3 (Items 5 and 6), and component 4 (Item 1).

### Reliability and Validation of PUTS

We calculated Cronbach's Alfa and Spearman-Brown coefficient based on the total sample, TS group, Younger group, and Older group. A total of 147 participants finished the 1-month retest, after which time we performed the test–retest reliability analysis. It was found that the test–retest reliability was 0.89. We performed the correlation analysis between the PUTS and YGTSS, CY-BOCS (Table 3). It was also found that the PUTS correlated significantly with the CY-BOCS and YGTSS in the TS group (*p* < 0.05). Furthermore, we performed the correlation analysis between subscales of YGTSS, CY-BOCS, and PUTS in the TS group. It was shown that PUTS correlated significantly with the motor tic symptoms in YGTSS (*p* < 0.001) and obsessive symptoms in CY-BOCS (*p* < 0.001) in the Younger TS group rather than the Older TS group (Table 4).

**Table 3:** Reliability and correlation analysis of PUTS.

**Reliability**	**Total sample *N* = 367**	**Younger group*N* = 230**	**Older group *N* = 137**	**TS group*N* = 252**
Cronbach's Alpha	0.753	0.762	0.731	0.755
Spearman-Brown	0.822	0.865	0.775	0.813
Correlation analysis between YGTSS and PUTS	0.180^*^	0.194	0.168	0.269^*^
Correlation analysis between CY-BOCS and PUTS	0.108	0.159	0.038	0.203^*^

*PUTS, Premonitory Urge for Tics Scale; YGTSS, Yale Global Tic Severity Scale; CY-BOCS, Children's Yale-Brown Obsessive Compulsive Scale*;

* *p < 0.05*.

**Table 4:** The Pearson correlation analysis between the subscales of YGTSS, CY-BOCS and PUTS in TS groups.

**Subscales of YGTSS or CY-BOCS**	**Motor Tic**	**Vocal Tic**	**Impairment**	**Compulsive symptoms**	**Obsessive symptoms**
TS Group (*N* = 252)	0.255^**^	0.083	0.138	0.055	0.245^**^
Younger TS Group (*N* = 154)	0.267^**^	0.061	0.150	0.012	0.266^**^
Older TS Group (*N* = 98)	0.184	0.087	0.038	0.070	0.156

*TS, Tourette Syndrome; PUTS, Premonitory Urge for Tics Scale; YGTSS, Yale Global Tic Severity Scale; CY-BOCS, Children's Yale-Brown Obsessive Compulsive Scale; Younger TS Group, age <10; Older TS Group, Age ≥ 10*;

** *p < 0.001*.

### Network Analysis of PUTS

**Figure 1:**
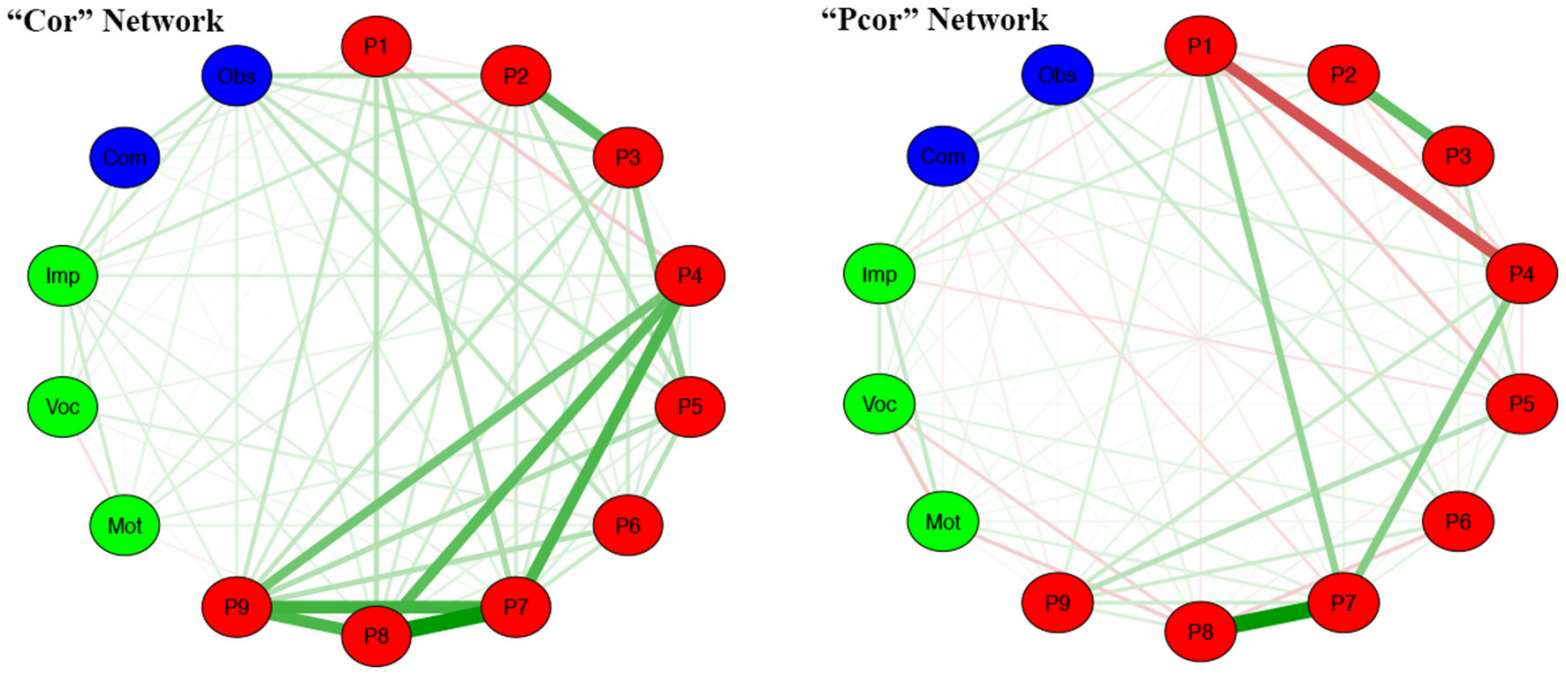
The “Cor” and “Pcor” networks of PUTS (P1–P9 means the nine items of PUTS; Obs, Obsession subscale of CY-BOCS; Com, Compulsive subscale of CY-BOCS; Imp, Impairment item of YGTSS; Voc, Vocal subscale of YGTSS; Mot, Motor subscale of YGTSS); Pcor, partial correlation; Cor, correlation; PUTS, Premonitory Urge for Tics Scale; YGTSS, Yale Global Tic Severity Scale; CY-BOCS, Children Yale-Brown Obsessive Compulsive Scale.

**Figure 2:**
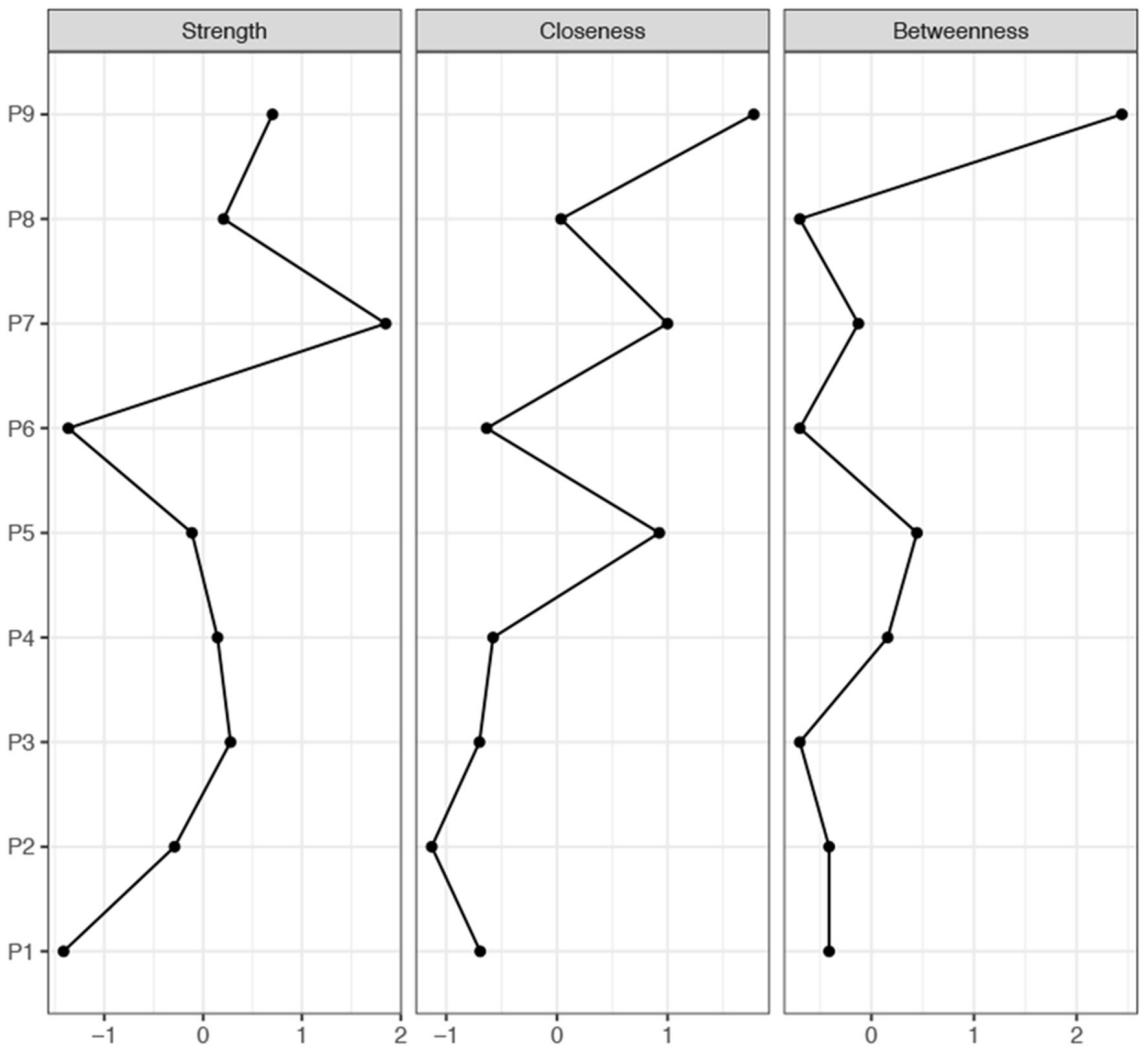
The strength, closeness, and betweenness of the “Cor” network of PUTS.

Both the “Cor” and “Pcor” network models were established. The “Cor” networks, including the nine items from PUTS, the impairment, severity of motor tic and vocal tic from the YGTSS, as well as the Obsession and Compulsion factors, from the CY-BOCS, were built based on the total sample. In general, for the obsessive-compulsive symptoms, PU was associated with the severity of obsessions symptoms in patients with tic disorders. In particular, for the items included in PUTS, a robust positive correlation was identified between Item 1 and Item 7, Item 7 and Item 8, Item 7 and Item 4, and Item 2 and Item 3, while there was a negative correlation between Item 1 and Item 4 (Figure 1). We also calculated the Strength, Closeness, and Betweenness of the network of nine-item PUTS. Figure 2 depicts the centrality plot of each node, showing that Item 7 had the highest Strength centrality, whereas Item 9 showed the highest Betweenness centrality and Closeness centrality.

## Discussion

This is the first study to explore the clinical characteristics of PU and its association with obsessive-compulsive symptoms and tic symptoms based on a large number of children with tic disorders in China. It has a great value to understand the characteristics of PU in Chinese people with tic disorders, especially in children with tic disorders. The main findings of this study included five aspects. First, we found that no difference in PU is identified in different age groups. Second, the results of the EFA showed the emergence of four primary factors of PUTS. Third, it showed good reliability and validation of the Chinese version of PUTS. Forth, it seemed that PU was associated with the severity of obsessions symptoms in patients with tic disorders. Last, the results of network analysis showed that Item 7 is a critical node for the PU, in addition to Items 1 and 4.

In the present study, we did not found the significance of PUs between the young and old groups, which was the same to previous studies. Woods et al. (2005) reported that the younger youths (mean = 18.3, SD = 4.6) did not differ significantly from older youths (mean = 18.6, SD = 7.3) in terms of mean PUTS score. Recently, Raines et al. (2018) also reported that younger participants (mean = 18.85, SD = 5.12) did not differ significantly from older participants (mean = 20.12, SD = 7.01) with respect to the mean PUTS score. However, a recent study that included a large sample (*N* = 656) reported that there was a significant difference between PU in different age groups. It indicated that PU was increased with age (Openneer et al., 2020). Ganos et al. (2015) suggested that interoceptive awareness was the strongest predictor of PUs in TS, with greater interoceptive awareness being associated with more urges. Another study reported that the specific interoceptive sensibility tends to increase with age (Nusser et al., 2020). Taken together, it indicated that, with the growing age, the ability of interoceptive awareness was growing, and more PU was identified. For the relationship between PU, interoceptive awareness and age, more evidence is needed to address this issue.

Furthermore, for the internal consistency of the PUTS, the age effect in PUTS is still inconsistent. For example, Woods et al. (2005) reported that Cronbach's alpha was much lower in the younger youths (0.57) than in the youths over 10 years (0.89). Raines et al. (2018) also reported the same results. However, Openneer et al. (2020) found that the Cronbach's alpha was at the same level in two young groups (aged 3–7 and aged 8–10, 0.84 and 0.83), but low Cronbach's alpha in the old group (aged 11–16, 0.76). In the present study, we also reported the same level in different age groups. We might need more evidence to confirm this inconsistency in the future.

For the structure of PU, four factors were identified in this present study. We found that Items 4, 7, 8, and 9 belonged to component 1, Items 2 and 3 belonged to component 2, Items 5 and 6 belonged to component 3, and Item 1 belonged to component 4. However, the results seemed not the same as recent studies about the structure of PUTS. For example, Raines et al. (2018) found a two-factor model with one factor capturing the quality of premonitory sensations while the other factor assessed the overall intensity of the urges. Openneer et al. (2020) reported a two-factor structure of the PUTS in children of 11 years and older, distinguishing between sensory phenomena related to tics and mental phenomena related to obsessive-compulsive disorder. Another study showed a three-factor model of the 10 items of the PUTS, one factor included the urge intensity, whereas the other two factors can be assumed to reflect the sensory aspect of urges and subjective aspect of control, respectively (Brandt et al., 2016). Overall, according to the previous study about the factors of PUTS, it indicated that there were at least four dimensions of PU, the first two are the intensity and frequency which reflect the severity of PU; the third one and fourth one are the “sensory” aspect and “mental” aspect of PU. However, a larger sample size and across culture studies might be needed to explore the different dimensions of PU.

The results of network analysis showed that Item 7 is a critical node for the PU, in addition to Items 1 and 4. Interestingly, a negative correlation was identified between Items 1 (about the feeling of “itchy”) and 4 (about the feeling “not just right”), indicating that these two feelings might not always be reported in the same patients. The feeling of “itchy” is more relevant to sensory feelings, while a feeling of “not just right” is more in the category of mental phenomena. Based on these results in this present study, it indicated that component 4 might be the “sensory feelings” aspect of PUs, and component 1 might be the “mental phenomena” aspect of PUs. The negative correlation between these two aspects suggests that these two types of PU (sensory feelings and mental phenomena) might exist in a “trade-off” relationship, but further exploration is needed in future studies. Furthermore, based on the network analysis, we found two subnetworks of PUs, one is component 1 (including Items 4, 7, 8, and 9), the others are components 2 and 3 (including Items 2, 3, and 5). Item 6 showed a weak correlation with other items. To our knowledge, there are few studies that focus on the items of PUTS; these results about the items will provide useful information for the development of PUTS in the future.

Our previous meta-analysis showed evidence that the PU showed strong correlations with tics (Wang et al., 2019). PU seemed to play a critical role in the expression of tic symptoms, but it can also be considered as an indicator of the severity of tic symptoms. Moreover, Habit Reversal Training (HRT) (Hwang et al., 2012; Piacentini and Chang, 2016; Viefhaus et al., 2020), as recommended by the American Academy of Neurology as the first-line therapy for TS (Pringsheim et al., 2019), notes that the most critical step of HRT is the awareness of PU (Seragni et al., 2018; Nissen et al., 2019b). Thus, we should pay more attention to PUs in the treatment research for tic disorders, especially for the assessments of PUs at the baseline and after HRT trials.

For the future development of PUTS, several issues need to be further addressed. First, it was reported that the PUTS demonstrated good reliability and validity in children and adolescents (Raines et al., 2018). However, many TS patients in China who are younger than 8 years find it difficult to read and understand the self-checklists, which may limit the utility of the instrument. Second, Brandt et al. (2016) developed a real-time urge monitor (RUM) to detect PUs, although it is found that the convergent validity between the PUTS and the real-time urge assessment monitor is good, it also suggested that PUTS might assess more than one dimension of urges, such as the intensity and frequency. Moreover, the locations of the PU on the body are not assessed on the PUTS, thus limiting the use of the PUTS in communicating effectively with the patient (Li et al., 2019). Finally, the impaired function, intensity, and frequency of PU should also be covered, which are important indicators for the severity of tic symptoms. Therefore, future development of PUTS should include further discussion of additional dimensions of PU.

This study has three limitations: first, we included only children and adolescents in our sample due to convenience, and a sample of adults with TS will be needed for future studies. Second, YGTSS and CY-BOCS were used as the measures of concurrent validity, but other scales (i.e., the University of São Paulo Sensory Phenomena Scale (USP-SPS)) used for the assessment of PUs, might be more suitable. Third, there was no examination of discriminant validity for PUTS in this study. Notwithstanding these limitations, this study demonstrates good psychometric properties of the Chinese version of the PUTS and suggests that this version is suitable for clinical and research purposes for the assessment of PUs in patients with tic disorders in Chinese settings. It also provides detailed information about the structure of PUTS, which will benefit the future development of PUTS.

## Conclusion

The Chinese version of PUTS showed good reliability and validity. It can be used in Chinese children and adolescent patients with tic disorders, especially in TS patients. Future research is warranted to investigate the age effect and factor structure of PU. Future development of the PUTS should focus on assessing more dimensions of urges, such as the intensity, frequency, “sensory” and “mental” aspects.

## Data Availability Statement

The original contributions presented in the study are included in the article/supplementary material, further inquiries can be directed to the corresponding author.

## Ethics Statement

The studies involving human participants were reviewed and approved by the Ethics Committee of Beijing Children's Hospital. Written informed consent to participate in this study was provided by the participants' legal guardian/next of kin.

## Author Contributions

For this manuscript, YC and YL took the initiative. YG, LY, JY, FWe, FWa, and JL finished the data collection. YL performed the data analysis and finished the draft. DW gave detailed suggestions to this article. All authors contributed to the article and approved the submitted version.

## Funding

This study was supported by the Special Fund of the Pediatric Medical Coordinated Development Center of Beijing Hospitals Authority, No. XTYB201802.

## Conflict of Interest

The authors declare that the research was conducted in the absence of any commercial or financial relationships that could be construed as a potential conflict of interest.

## Publisher's Note

All claims expressed in this article are solely those of the authors and do not necessarily represent those of their affiliated organizations, or those of the publisher, the editors and the reviewers. Any product that may be evaluated in this article, or claim that may be made by its manufacturer, is not guaranteed or endorsed by the publisher.

## References

[B1] Banaschewski T. Woerner W. Rothenberger A. (2003). Premonitory sensory phenomena and suppressibility of tics in Tourette syndrome: developmental aspects in children and adolescents. Dev. Med. Child Neurol. 45, 700–703. 10.1111/j.1469-8749.2003.tb00873.x 14515942

[B2] Beaton D. E. Bombardier C. Guillemin F. Ferraz M. B. (2000). Guidelines for the process of cross-cultural adaptation of self-report measures. Spine 25, 3186–3191. 10.1097/00007632-200012150-00014 11124735

[B3] Brandt V. C. Beck C. Sajin V. Anders S. Munchau A. (2016). Convergent validity of the PUTS. Front. Psychiatry 7:51. 10.3389/fpsyt.2016.00051 27092085 PMC4823310

[B4] Conceicao V. A. Dias A. Farinha A. C. Maia T. V. (2017). Premonitory urges and tics in Tourette syndrome: computational mechanisms and neural correlates. Curr. Opin. Neurobiol. 46, 187–199. 10.1016/j.conb.2017.08.009 29017141

[B5] Contreras A. Nieto I. Valiente C. Espinosa R. Vazquez C. (2019). The study of psychopathology from the network analysis perspective: a systematic review. Psychother. Psychosom. 88, 71–83. 10.1159/000497425 30889609

[B6] Cox J. H. Seri S. Cavanna A. E. (2018). Sensory aspects of Tourette syndrome. Neurosci. Biobehav. Rev. 88, 170–176. 10.1016/j.neubiorev.2018.03.016 29559228

[B7] Dambi J. M. Corten L. Chiwaridzo M. Jack H. Mlambo T. Jelsma J. (2018). A systematic review of the psychometric properties of the cross-cultural translations and adaptations of the Multidimensional Perceived Social Support Scale (MSPSS). Health Qual. Life Outcomes 16:80. 10.1186/s12955-018-0912-0 29716589 PMC5930820

[B8] Epskamp S. Borsboom D. Fried E. I. (2018). Estimating psychological networks and their accuracy: a tutorial paper. Behav. Res. Methods 50, 195–212. 10.3758/s13428-017-0862-1 28342071 PMC5809547

[B9] Epskamp S. Cramer A. O. Waldorp L. J. Schmittmann V. D. Borsboom D. (2012). qgraph: network visualizations of relationships in psychometric data. J. Stat. Softw. 48, 1–18. 10.18637/jss.v048.i04

[B10] Ganos C. Garrido A. Navalpotro-Gomez I. Ricciardi L. Martino D. Edwards M. J.. (2015). Premonitory urge to tic in Tourette's is associated with interoceptive awareness. Mov Disord. 30, 1198–1202. 10.1002/mds.26228 25879819

[B11] Hwang G. C. Tillberg C. S. Scahill L. (2012). Habit reversal training for children with Tourette syndrome: update and review. J. Child Adolescent Psychiatric Nurs. 25, 178–183. 10.1111/jcap.12002 23121140

[B12] Kyriazi M. Kalyva E. Vargiami E. Krikonis K. Zafeiriou D. (2019). Premonitory urges and their link with tic severity in children and adolescents with tic disorders. Front. Psychiatry 10:569. 10.3389/fpsyt.2019.00569 31474885 PMC6702331

[B13] Leckman J. F. Riddle M. A. Hardin M. T. Ort S. I. Swartz K. L. Stevenson J.. (1989). The Yale Global Tic Severity Scale: initial testing of a clinician-rated scale of tic severity. J. Am. Acad. Child Adolesc. Psychiatry 28, 566–573. 10.1097/00004583-198907000-00015 2768151

[B14] Leckman J. F. Walker D. E. Cohen D. J. (1993). Premonitory urges in Tourette's syndrome. Am. J. Psychiatry 150, 98–102. 10.1176/ajp.150.1.98 8417589

[B15] Li Y. Zhang J. S. Wen F. Lu X. Y. Yan C. M. Wang F.. (2019). Premonitory urges located in the tongue for tic disorder: two case reports and review of literature. World J Clin Cases. 7(12):1508–1514. 10.12998/wjcc.v7.i12.1508 31363480 PMC6656667

[B16] Li F. Cui Y. Li Y. Guo L. Ke X. Liu J.. (2021). Prevalence of mental disorders in school children and adolescents in China: diagnostic data from detailed clinical assessments of 17,524 individuals. J. Child Psychol. Psychiatry. 10.1111/jcpp.13445 34019305

[B17] McGuire J. F. Piacentini J. Storch E. A. Murphy T. K. Ricketts E. J. Woods D. W.. (2018). A multicenter examination and strategic revisions of the Yale Global Tic Severity Scale. Neurology 90, e1711–e1719. 10.1212/WNL.0000000000005474 29653992 PMC5952973

[B18] Nissen J. B. Kaergaard M. Laursen L. Parner E. Thomsen P. H. (2019b). Combined habit reversal training and exposure response prevention in a group setting compared to individual training: a randomized controlled clinical trial. Euro. Child Adolesc. Psychiatry 28, 57–68. 10.1007/s00787-018-1187-z 29956034 PMC6349803

[B19] Nissen J. B. Parner E. T. Thomsen P. H. (2019a). Predictors of therapeutic treatment outcome in adolescent chronic tic disorders. BJPsych Open 5:e74. 10.1192/bjo.2019.56 31409430 PMC6737514

[B20] Nusser L. Pollatos O. Zimprich D. (2020). Age-related effects on interoceptive accuracy, general interoceptive sensibility, and specific interoceptive sensibility. Euro. J. Health Psychol. 27, 154–170. 10.1027/2512-8442/a000060

[B21] Openneer T. J. C. Tarnok Z. Bognar E. Benaroya-Milshtein N. Garcia-Delgar B. Morer A.. (2020). The Premonitory Urge for Tics Scale in a large sample of children and adolescents: psychometric properties in a developmental context. An EMTICS study. Eur. Child Adolesc. Psychiatry 29, 1411–1424. 10.1007/s00787-019-01450-1 31802271 PMC7501098

[B22] Piacentini J. Chang S. (2016). Habit reversal training for tic disorders in children and adolescents. Behav. Modif. 29, 803–822. 10.1177/0145445505279385 16204417

[B23] Pringsheim T. Okun M. S. Muller-Vahl K. Martino D. Jankovic J. Cavanna A. E.. (2019). Practice guideline recommendations summary: treatment of tics in people with Tourette syndrome and chronic tic disorders. Neurology 92, 896–906. 10.1212/WNL.0000000000007466 31061208 PMC6537133

[B24] Rae C. L. Larsson D. E. O. Garfinkel S. N. Critchley H. D. (2019). Dimensions of interoception predict premonitory urges and tic severity in Tourette syndrome. Psychiatry Res. 271, 469–475. 10.1016/j.psychres.2018.12.036 30544073

[B25] Raines J. M. Edwards K. R. Sherman M. F. Higginson C. I. Winnick J. B. Navin K.. (2018). Premonitory Urge for Tics Scale (PUTS): replication and extension of psychometric properties in youth with chronic tic disorders (CTDs). J. Neural. Transm. 125, 727–734. 10.1007/s00702-017-1818-4 29185077

[B26] Scahill L. Riddle M. A. McSwiggin-Hardin M. Ort S. I. King R. A. Goodman W. K.. (1997). Children's Yale-Brown Obsessive Compulsive Scale: reliability and validity. J. Am. Acad. Child Adolesc. Psychiatry 36, 844–852. 10.1097/00004583-199706000-00023 9183141

[B27] Sellbom M. Tellegen A. (2019). Factor analysis in psychological assessment research: common pitfalls and recommendations. Psychol. Assess. 31, 1428–1441. 10.1037/pas0000623 31120298

[B28] Seragni G. Chiappedi M. Bettinardi B. Zibordi F. Colombo T. Reina C.. (2018). Habit reversal training in children and adolescents with chronic tic disorders: an Italian randomized, single-blind pilot study. Minerva Pediatr. 70, 5–11. 10.23736/S0026-4946.16.04344-9 26583455

[B29] Storch E. A. Murphy T. K. Adkins J. W. Lewin A. B. Geffken G. R. Johns N. B.. (2006). The children's Yale-Brown obsessive-compulsive scale: psychometric properties of child- and parent-report formats. J Anxiety Disord. 20, 1055–1070. 10.1016/j.janxdis.2006.01.006 16503111

[B30] Viefhaus P. Feldhausen M. Gortz-Dorten A. Volk H. Dopfner M. Woitecki K. (2020). Efficacy of habit reversal training in children with chronic tic disorders: a within-subject analysis. Behav. Modif. 44, 114–136. 10.1177/0145445518796203 30146896

[B31] Wang F. Li Y. Liu J. Wen F. Yan C. Zhang J.. (2019). The correlation between the severity of premonitory urges and tic symptoms: a meta-analysis. J Child Adolesc. Psychopharmacol. 29, 652–658. 10.1089/cap.2019.0048 31343266

[B32] Wen F. Gu Y. Yan J. Liu J. Wang F. Yu L.. (2021). Revisiting the structure of the Yale Global Tic Severity Scale (YGTSS) in a sample of Chinese children with tic disorders. BMC Psychiatry. 21, 394. 10.1186/s12888-021-03399-5 34372795 PMC8351146

[B33] Woods D. W. Piacentini J. Himle M. B. Chang S. (2005). Premonitory Urge for Tics Scale (PUTS): initial psychometric results and examination of the premonitory urge phenomenon in youths with Tic disorders. J. Dev. Behav. Pediatr. 26, 397–403. 10.1097/00004703-200512000-00001 16344654

